# Validation of a Markerless Multi-Camera Pipeline for Bouldering Fall Kinematics

**DOI:** 10.3390/s26020662

**Published:** 2026-01-19

**Authors:** Nathan Carretier, Erwan Beurienne, Marie-Hélène Beauséjour, Lucas Gros, Claire Bruna-Rosso, Marine Dorsemaine, Michel Behr, Nicolas Bailly, Julien Clément

**Affiliations:** 1 Department of Systems Engineering, École de Technologie Supérieure, Montréal, QC H3C 1K3, Canada; nathan.carretier.1@ens.etsmtl.ca (N.C.); marie-helene.beausejour@etsmtl.ca (M.-H.B.); lucas.gros.1@ens.etsmtl.ca (L.G.); 2 Laboratoire de Biomécanique Appliquée, Aix-Marseille Université, Université Gustave Eiffel, 13015 Marseille, France; erwan.beurienne@univ-eiffel.fr (E.B.); claire.bruna-rosso@univ-eiffel.fr (C.B.-R.); marine.dorsemaine@univ-eiffel.fr (M.D.); michel.behr@univ-eiffel.fr (M.B.); nicolas.bailly@univ-eiffel.fr (N.B.); 3 Tyyny, Context’, 42500 Le Chambon Feugerolles, France; 4 Research Center, CIUSSS Nord de l’île de Montréal, Montréal, QC H4J 1C5, Canada; 5 Institut National du Sport du Québec, Montréal, QC H1V 3N7, Canada

**Keywords:** bouldering, fall, inertial measurement unit, markerless, video analysis, Pose2Sim, kinematics, acceleration, injury prevention, sports biomechanics

## Abstract

**Highlights:**

**What are the main findings?**

- Pose2Sim provided valid estimates of fall height and peak velocity during bouldering falls compared with 2D video analysis.
- Pose2Sim systematically underestimated peak acceleration compared with high-frequency IMUs, especially at the ankle and sacrum.

**What is the implication of the main finding?**

- Markerless video analysis is appropriate for studying fall kinematics and typology in indoor bouldering.
- IMUs remain necessary to quantify impact intensity, and future work should explore the combination of both IMUs and video to overcome this limitation.

**Abstract:**

Indoor bouldering is a popular and rapidly growing sport in which climbers fall repeatedly from walls up to 4–5 m high, making lower-limb injuries common. It is therefore essential to understand fall kinematics and impact conditions, yet fall kinematics remain poorly documented because laboratory motion capture is impractical in gyms. This study aimed to validate a markerless multi-camera pipeline (Pose2Sim) against a 2D video annotation tool (Kinovea) for displacement and velocity measurement, and against IMUs for peak acceleration. Ten teenage athletes (3 males, 7 females; 14–17 years) performed 40 falls recorded with five cameras (GoPro HERO12, USA, 2.7 K, 240 fps) and three IMUs (Blue Trident, Vicon, UK; ±200 g, 1600 Hz). Cut-off frequencies were set using Yu’s method (13 Hz for video, 39 Hz for IMUs). Pose2Sim’s results closely matched those of Kinovea for fall height and peak velocity with non-significant differences but underestimated peak acceleration. At the forehead, no significant difference was found, likely due to smaller accelerations at the head. Markerless video analysis is appropriate for studying fall kinematics and typology in indoor bouldering. IMUs remain necessary to quantify impact intensity, and future work should explore the combination of both IMUs and video to overcome this limitation.

## 1. Introduction

Indoor bouldering has become one of the fastest-growing climbing disciplines, practiced both recreationally and competitively worldwide [1]. Falls from wall heights of up to 4–5 m are inherent to this activity and, despite the presence of crash pads, remain the leading cause of traumatic injuries [1,2]. Recent evidence further indicates that the design of landing pads (made of foam layers, covers and trapped air) can substantially affect their protective capacity [3,4]. Epidemiological studies report injury rates around 2.7 ± 4.5 per 1000 h of practice [2], with lower limbs injuries like ankle or knee sprains and fractures being the most frequent outcomes [1]. Severe cases, such as complex knee ligament injuries, have also been reported in bouldering [5]. As participation increases, the need for objective data on fall mechanisms and impact characteristics is becoming increasingly important for prevention and for guiding safe training practices.

A recent study has proposed a taxonomy of bouldering falls, classifying them according to initial posture, body rotation during the fall, and landing position. This framework aimed to establish a link between fall kinematics and injury patterns, including injury type and location. While this study suggests that different fall mechanisms (such as vertical versus leaning initial postures) lead to distinct injury profiles, the findings remain largely qualitative and quantifying fall kinematics in gym settings remains challenging [6].

Two-dimensional (2D) video analysis software Kinovea 2023.1.1 (kinovea.org) is a manual video annotation and motion analysis tool that enables frame-by-frame measurements of displacement and velocity and has shown acceptable validity for planar movements and simple sports tasks, such as gait analysis, jump performance, and basic repetitive movements in activities like walking or running [7,8,9]. More recently, it was also applied to fall reconstruction scenarios, for example, in experimental e-scooter crashes, where it provided reliable estimates of head displacement and velocity [10]. These applications highlight its usefulness for global kinematic assessment, yet the software remains limited for high-frequency events such as impact accelerations [7,9]. Moreover, this 2D approach is unsuitable for capturing movements occurring out of the camera plane, which is particularly problematic during bouldering falls. Inertial Measurement Units (IMUs) are widely used in biomechanics to capture high-frequency accelerations [10,11,12,13,14]. They are highly relevant for quantifying impact intensity but only provide local measurements at the sensor site, while also being subject to soft-tissue artifacts. In addition, because IMUs must be directly attached to the athlete, they may cause discomfort or interfere with movement, and their use is impractical in official competitions.

Recent advances in markerless motion capture, and particularly multi-camera pipelines such as Pose2Sim (P2S) [15,16], offer a promising compromise. These methods use computer vision and deep learning to reconstruct three-dimensional (3D) kinematics from synchronized videos, making them useful for applications in sports science and injury biomechanics. Previous validations of P2S focused mainly on cyclic movements such as gait (spatiotemporal parameters and joint angles), cycling (segment kinematics), and simple repetitive tasks, all assessed under controlled laboratory conditions [17,18,19,20]. However, its accuracy under rapid and high-impact conditions such as bouldering falls remains largely unexplored. In these situations, 360° camera placement is impossible because the climbing wall blocks rear viewpoints. In particular, the ability of P2S to capture both global kinematics (fall height, velocity) and local impact dynamics (peak acceleration at the ankle, sacrum, and forehead) was not yet evaluated in a climbing context.

The purpose of this study was therefore to validate P2S for the analysis of bouldering falls in a real climbing gym environment. Specifically, we tested whether P2S provides displacement and velocity measures comparable to a 2D video analysis software reference (Kinovea) and whether its acceleration estimates are consistent with high-frequency IMU recordings. In addition, we assessed the morphological fidelity of P2S by comparing reconstructed segment lengths with direct anthropometric measurements. Unlike most prior P2S validations performed on cyclic tasks in controlled laboratory settings, the present work evaluates P2S in a real-condition indoor bouldering context characterized by high-impact, non-cyclic falls and rotations. Importantly, this context also imposes constrained camera viewpoints (no full 360° coverage due to the wall and gym layout), which differs from typical multi-camera laboratory configurations. This study therefore extends P2S validation to a practical field setting and explicitly delineates what video-based kinematics can reliably estimate (global fall kinematics) versus what remains limited for impact quantification (peak accelerations) without high-frequency IMUs.

## 2. Materials and Methods

### 2.1. Participants and Ethics

From a larger cohort of twenty-two athletes recorded, ten teenage athletes (3 males, 7 females; age 14–17 years) were selected for this specific analysis (detailed in Section 2.6). All participants had ≥3 years of bouldering experience and reported no recent lower-limb injury. Written informed consent was obtained from each athlete and, because all were minors, from their parents or legal guardians. The study was approved by the École de Technologie Supérieure (Montréal, QC, Canada) ethics committee (H20240603).

### 2.2. Anthropometric Data Collection

Anthropometric data were collected before the experimental setup to characterize the participants and to assess the morphological fidelity of P2S segment lengths. Stature, limb lengths (leg, tibia, trunk, arm, forearm, foot), wingspan, foot width, and segmental circumferences (thigh, calf, arm, forearm, trunk) were obtained with a flexible tape according to the International Standards for Anthropometric Assessment [21]. These measures were recorded once per athlete by the same investigator and later compared to segment lengths reconstructed by P2S.

### 2.3. Experimental Setup

**Figure 1:**
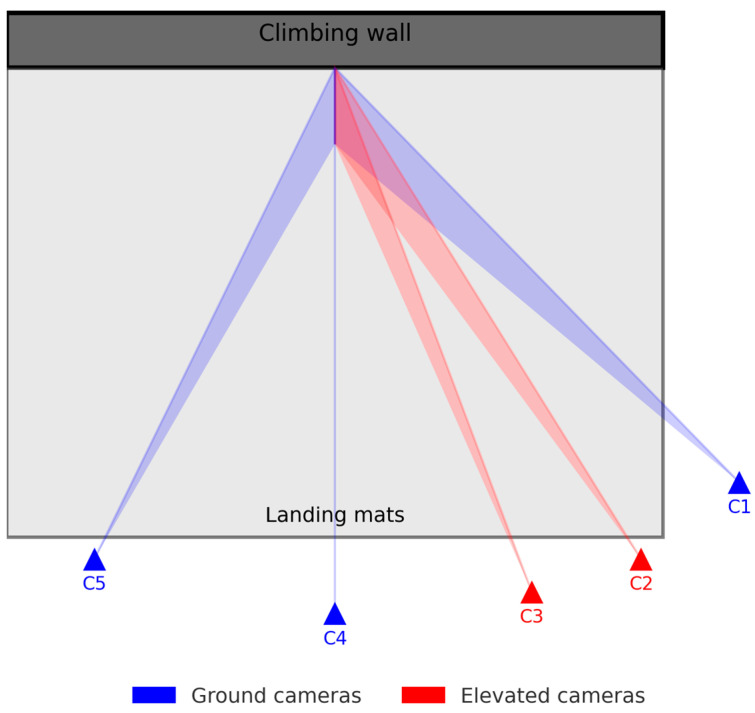
Camera setup and field-of-view coverage in the bouldering gym (blue = ground; red = elevated by 5m).

The experiments took place in a commercial climbing gym with walls up to 4.5 m high and thick crash pads covering the floor. Five cameras (HERO12 Black, GoPro Inc., San Mateo, CA, USA) were installed around the wall to maximize visibility of the athletes and minimize occlusions. The cameras were labeled C1–C5 from right to left in the setup (Figure 1), and each camera was placed at the same location across all recording sessions to facilitate subsequent data processing. Videos were recorded at 240 fps with a maximum resolution of 2.7 K. For Kinovea, we used the frontal view (C4) because it provided the most orthogonal perspective of the athlete during the T-pose and the fall, thereby minimizing perspective distortion when scaling and extracting planar kinematics, as shown in Figure 1.

**Figure 2:**
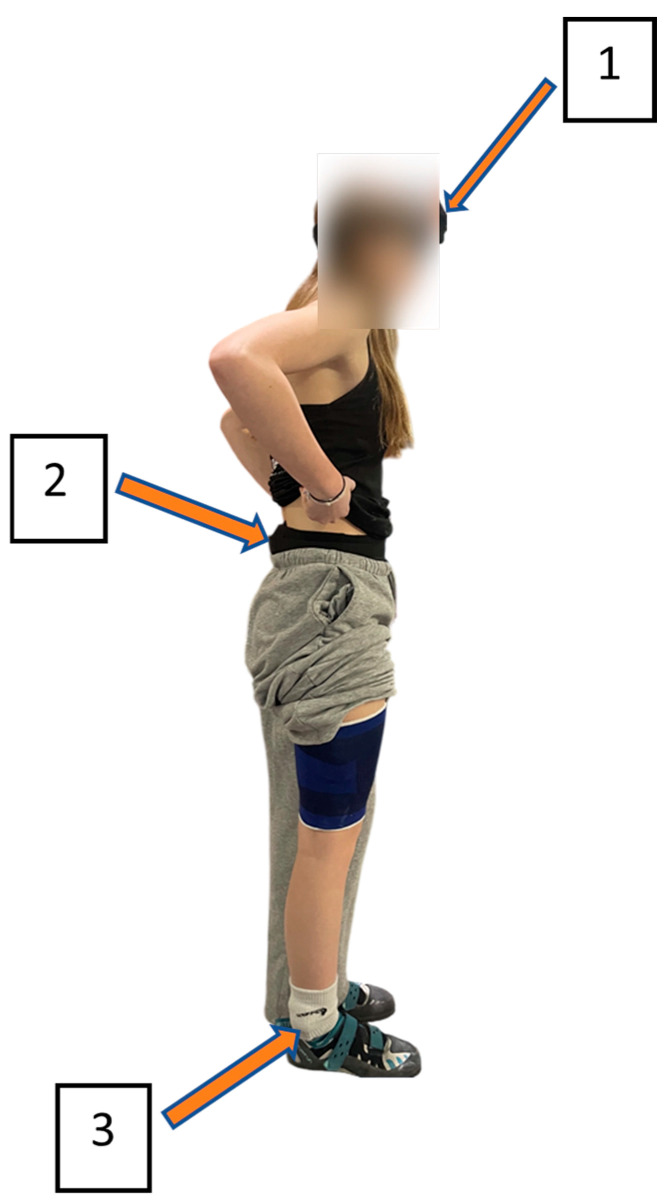
IMU position on the participant: forehead = 1, sacrum = 2, and ankle = 3.

Three IMUs (Blue Trident, Vicon Motion Systems Ltd., Oxford, UK) [13,14] were attached to the right ankle, sacrum, and forehead. Triaxial acceleration was sampled at 1600 Hz with a measurement range of ±200 g. Sensors were fixed with elastic straps and hypoallergenic tape, with gel pads placed underneath to reduce discomfort and absorb potential shocks in case of direct contact. The placement of the IMUs on the participant is shown in Figure 2.

Analyses were conducted focusing on three anatomically relevant sites. The ankle was chosen because it is the most distal lower-limb segment and a common site of injury during bouldering falls [1]. The sacrum was selected as a proxy for the trunk and center of mass, given its central location in the body and its role in energy transmission during impacts [22]. Finally, the forehead was included to assess head exposure, as it represents a critical area in terms of injury risk during high-impact events. This methodological choice is consistent with the literature that employs inertial sensors to characterize head motion and associated risks [23].

These three locations correspond directly to identifiable P2S joint markers (ankle, sacrum/hip region, and head), allowing consistent comparison between video-based and sensor-based measurements.

**Figure 3:**
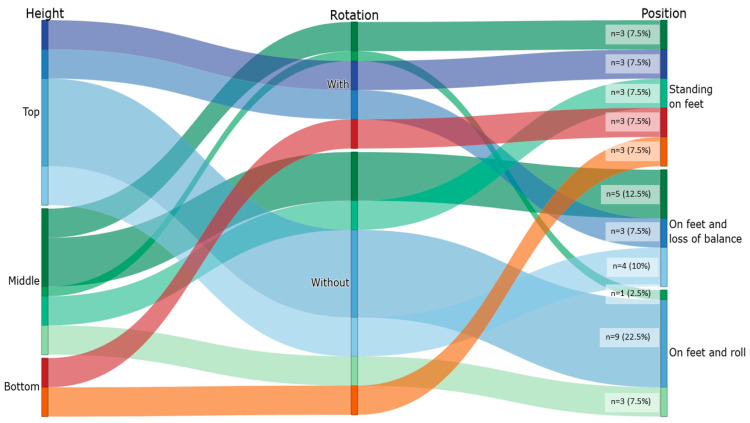
Kinematics of bouldering falls scenario map. Parallel-categories diagram linking start height (Top/Middle/Bottom) → body rotation (With/Without) → position at reception (Standing on feet; On feet with loss of balance; On feet and roll). Flow widths are proportional to counts (n = 40).

Twenty-two athletes performed a total of over 200 falls. From this dataset, we retained a purposeful subset of 10 athletes and extracted 40 trials for analysis (12 natural falls and 28 controlled jumps). Trial selection aimed to (i) provide adequate statistical power for method-comparison analyses, (ii) balance fall scenarios (start height, presence/absence of rotation, and landing outcome), and (iii) ensure sufficient data quality for synchronized multi-camera reconstruction and IMU processing (e.g., minimal additional occlusions such as bystanders obstructing camera views, adequate visibility for 3D reconstruction, and complete IMU recordings) as detailed in Section 2.6. The different fall types were intentionally chosen to capture a range of kinematic behaviors, including varying fall heights (top, middle, bottom), the presence of body rotations during the fall (yes or no), and reception positions (standing on feet, on feet and roll, on feet and loss of balance). This approach aimed to provide a robust dataset for the analysis while accounting for the variability inherent in real-world climbing situations. The fall scenarios are illustrated in Figure 3, which presents a map linking the start height, the presence or absence of a rotation, and the position at reception. Across the 40 selected trials, start heights were distributed as Top (n = 19), Middle (n = 15), and Bottom (n = 6). Rotations were observed in 13 trials (Without rotation: n = 27). Reception positions were “Standing on feet” (n = 15), “On feet with loss of balance” (n = 12), and “On feet and roll” (n = 13), as summarized in Figure 3. Trials were embedded in the athletes’ regular training sessions, after a warm-up supervised by their coach. Each athlete climbed at their own rhythm, choosing routes freely as they were asked to climb as they usually do. Investigators did not intervene to preserve ecological validity. Between trials, recordings were paused to allow athletes to rest and prepare without time pressure.

### 2.4. Calibration and Synchronization

Intrinsic camera parameters were calibrated using a checkerboard, while a 3D cube (1.0 × 1.5 m) was used to perform extrinsic calibration of the capture volume [24]. IMUs were calibrated before each session with figure-eight motions and flat rotations, followed by a stabilization phase [14].

Before each climb a standardized sequence was performed by the athlete: a vertical jump, a T-pose, and a single hand clap. Camera recordings were initiated using a wireless remote connected to all cameras, which can introduce small differences in recording start times across views. Therefore, synchronization between cameras was performed using the hand clap event. First, the exact frame of hand contact was identified in each camera view using Kinovea (frame-by-frame). The camera for which the clap occurred earliest was used as the reference (and corresponded to the shortest recording, as all cameras captured the same scene with only a small trigger delay). For each other camera, the temporal offset relative to the reference was computed, and the beginning of the video was trimmed by the corresponding amount so that the clap was aligned across all five views. After trimming the beginning of each video based on the hand-clap contact frame identified in Kinovea, we verified that the residual inter-camera delay was ≤1 frame at 240 fps (≤4.17 ms) by comparing the first and last frames as well as the total number of frames of each trimmed video, confirming that all views covered the same time window.

Synchronization between video and IMU data was achieved using the vertical jump and T-pose of each athlete before the climb since these events were clearly identifiable in video recordings and produced distinct acceleration peaks and a stable, low-acceleration phase in IMU signals, ensuring precise temporal alignment.

For Kinovea calibration, each athlete performed a T-pose facing the frontal camera (C4 in Figure 1); anthropometric dimensions (e.g., stature, wingspan) were then used to scale the video plane.

### 2.5. Data Processing

#### 2.5.1. Pose2Sim

Videos were processed with Pose2Sim v0.10.20 [15,16,24], which performs multi-camera markerless Three-dimensional reconstruction of joint trajectories. Pose estimation was performed with RTMLib using the Body_with_feet model in performance mode, and a single-person configuration (multi_person = false). Person association followed a single-person workflow (tracked keypoint: Neck) with an association reprojection-error threshold of 20 px and a keypoint confidence (likelihood) threshold of 0.3. 3D joint trajectories were reconstructed by triangulation using at least two cameras (minimum cameras for triangulation = 2), with a triangulation likelihood threshold of 0.3 and a triangulation reprojection-error threshold of 25 px. Pose2Sim does not include an explicit joint-point optimization; joint trajectories result from multi-view triangulation under the above confidence and reprojection constraints. Missing data were linearly interpolated only for gaps shorter than 10 frames; larger gaps were left as missing values (filled with NaN). All parameters were defined in the project configuration file and applied consistently across all trials.

OpenSim inverse kinematics was not used as we stopped at the 3D reconstruction of the falls and did not import these trajectories into a musculoskeletal model [25,26]. From the reconstructed trajectories, fall height (vertical displacement), peak vertical velocity (z-axis), and peak acceleration norm were computed at three anatomical sites: ankle, sacrum, and forehead. In addition, segment lengths were extracted to assess morphological fidelity.

#### 2.5.2. Kinovea

Kinovea v0.9.5 [7,8,9] was used as a 2D reference for fall height and peak velocity. Tracking was semi-automatic; the operator manually selected the location corresponding to the sensor (e.g., ankle, sacrum, forehead) and Kinovea propagated the tracking across frames. The operator visually verified and corrected trajectories when occlusions or drifts occurred.

Displacement–time and velocity–time data, as well as estimated segment lengths, were exported as CSV files.

#### 2.5.3. IMUs

IMU data were recorded at 1600 Hz and exported with Capture.U. Since IMUs measure accelerations in local axes that are not directly comparable to global kinematics from P2S and Kinovea, the resultant acceleration norm was computed. Peak acceleration was defined as the maximum value during the impact phase for each sensor location (ankle, sacrum, forehead).

To investigate the specific contribution of sampling frequency to the observed discrepancies between video-based methods and IMUs, a resampling analysis was performed. Raw IMU data (1600 Hz) were downsampled to match the video frame rate (240 Hz) [27,28,29] by generating a uniform time vector starting from the first IMU timestamp and linearly interpolating the raw accelerations to these new time points. This resampling analysis allowed for a direct comparison of temporal resolution effects, while maintaining identical sensor locations and measurement conditions.

#### 2.5.4. Filtering

P2S and IMUs data were processed with a 4th-order zero-phase Butterworth filter. Cut-off frequencies were objectively selected with Yu’s residual method [30,31], yielding 13 Hz for video trajectories and 39 Hz for IMUs sampled at 1600 Hz. These values balanced noise reduction with preservation of high-frequency impact dynamics, consistent with biomechanics recommendations [32,33]. In this study, Kinovea was used with its default “Smooth” option, which applies a zero-phase, 2nd-order Butterworth low-pass filter to displacement trajectories. The software proposes an automatic cutoff frequency for each trajectory based on a residual-analysis criterion [34]. Prior to resampling to 240 Hz, an explicit anti-aliasing low-pass filter was applied (4th-order zero-phase Butterworth, 110 Hz cutoff), selected conservatively below the Nyquist frequency of the target sampling rate (120 Hz). The anti-aliased signals were then resampled to 240 Hz. Yu’s residual method was subsequently applied on the resampled signals to determine the biomechanically relevant low-pass cutoff for smoothing; this yielded ~13 Hz, which was then applied to the 240 Hz IMU signals [30,31].

### 2.6. Variables and Statistical Analysis

Normality of residuals was verified using the Shapiro–Wilk test before applying parametric tests. Sample size was determined using G*Power 3.1.9.7 software [35]. For paired *t*-tests comparing P2S vs. Kinovea measurements, a power analysis was conducted. A medium effect size (Cohen’s d = 0.5) was selected according to Cohen’s conventional benchmarks [36]. The significance level was set at α = 0.05, and a statistical power of 0.85 was chosen, which exceeds the commonly recommended minimum of 0.80 for balancing Type I and Type II error risks [37]. This yielded a required sample size of 31 trials. To ensure adequate statistical power for the repeated-measures ANOVA (RM-ANOVA) comparing three methods (P2S, Kinovea, IMU-based), the sample size was increased to 40 sequences, providing enough valid trials for robust analysis. To assess the agreement between P2S and Kinovea, intraclass correlation coefficients (ICC (2,1)) and Bland–Altman analyses were computed for both displacement and velocity at the ankle, sacrum, and forehead [38].

The main outcome variables were:

- Segment lengths (cm): P2S vs. anthropometric measurements.
- Fall height (m): P2S vs. Kinovea.
- Peak velocity (m·s^−1^): P2S vs. Kinovea.
- Peak acceleration (m·s^−2^): P2S vs. IMUs (ankle, sacrum, forehead).
- Peak acceleration width (ms): P2S vs. IMUs (ankle, sacrum, forehead).

For video-based methods (P2S and Kinovea), velocity was obtained by differentiating position trajectories over time, and acceleration was then obtained by differentiating velocity over time (i.e., acceleration is the second derivative of position).

Segment lengths were computed as joint-to-joint distances and averaged across frames for each trial. The analysis was performed twice: (1) after removing extreme values (>1000% relative error) corresponding to a tenfold increase relative to the expected segment length and indicative of clear pose estimation failures due to incorrect joint identification rather than intrinsic segment length estimation errors and (2) including all data points to examine how fall condition (Standing, Loss of Balance, Roll) influences reconstruction variability. Both results are reported in a single table to distinguish typical performance from condition-dependent tracking errors.

Fall Height was defined as the vertical displacement between take-off and the lowest point of the fall.

Peak velocity was defined as the maximum vertical velocity recorded between take-off and impact for each anatomical site.

Peak acceleration was defined as the maximum acceleration reached between take-off and impact for each anatomical site.

Peak acceleration width was defined as the time interval during which acceleration remained above 90% of P2S acceleration peak. The same absolute threshold (90% of the P2S peak, in m·s^−2^) was then applied to the IMU norm.

This threshold was selected to quantify the duration of the main impact transient (near-peak region) while reducing sensitivity to lower-amplitude post-impact oscillations and baseline noise. Because the biomechanical and injury-related relevance of this metric in bouldering is not yet established, peak acceleration width is reported here as an exploratory descriptor of impact sharpness. A 90% threshold was suggested here as a pragmatic compromise: narrow enough to focus on the near-peak transient, yet wide enough to remain robust to sampling resolution and small peak-shape variations.

IMU peak acceleration was quantified using the resultant acceleration norm (‖a‖). Directional components (e.g., vertical acceleration) were not retained because IMU signals are expressed in a local sensor frame that may rotate during the fall and landing. The norm therefore provides a robust, orientation-independent indicator of impact intensity, at the cost of losing directional information.

Analyses were conducted at the trial (fall) level. Although multiple falls were recorded per participant, mixed-effects or repeated-measures models were not applied due to the limited number of participants (n = 10), the unbalanced number of falls per participant (1–6), and the non-repetitive nature of fall events. The primary objective of the analyses was to compare measurement methods at the level of individual falls rather than to model participant-specific effects.

## 3. Results

### 3.1. Morphological Fidelity

Segment lengths reconstructed by P2S showed good overall morphological fidelity when all 40 falls were included with and without outliers (Table 1). Across the ten segments, mean relative errors ranged from −0.5% (right shank) to +8.9% (left upper arm), with standard deviations between ~15% and 38%. When examined separately by fall scenario (Standing, Loss of Balance, Roll), paired t-tests of segment-length relative errors did not reveal any systematic bias for any segment (all *p* > 0.05), both when including and excluding extreme values. Specifically, *p*-values ranged from 0.16 to 0.93 in Standing, 0.11–0.93 in Loss of Balance, and 0.08–0.84 in Roll. These results indicate that fall posture did not significantly affect mean reconstruction accuracy, while Roll trials primarily increased variability and the occurrence of extreme tracking failures (Table 1).

**Figure 4:**
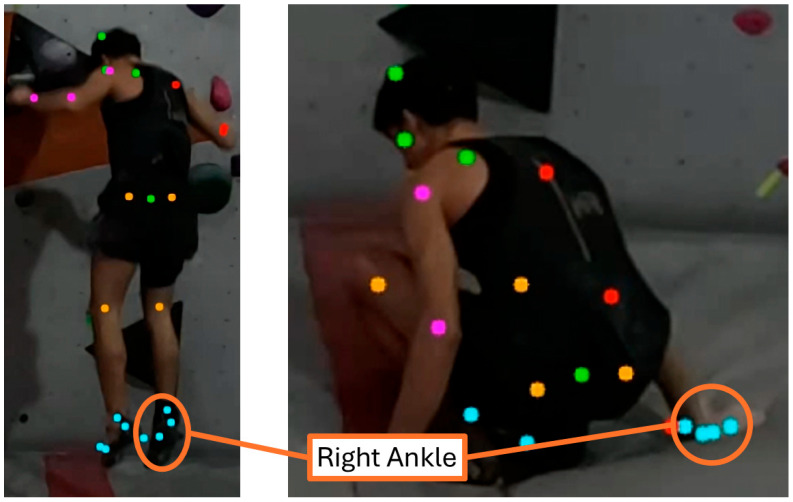
Pose estimation quality comparison during a bouldering fall sequence. (**Left**) Airborne phase showing clear joint visibility and accurate pose detection across all body segments. (**Right**) Impact phase where ankle penetration into the crash mat surface and impact-induced body deformation creates multiple tracking challenges. Colored dots represent detected key points from the pose estimation algorithm (ankle errors highlighted in orange).

However, when extreme deviations were retained, variability increased markedly, especially during Roll trials. Standing and Loss of Balance trials displayed comparatively fewer extreme estimates overall, although these deviations are still visible in Figure 4.

### 3.2. Fall Height

**Figure 5:**
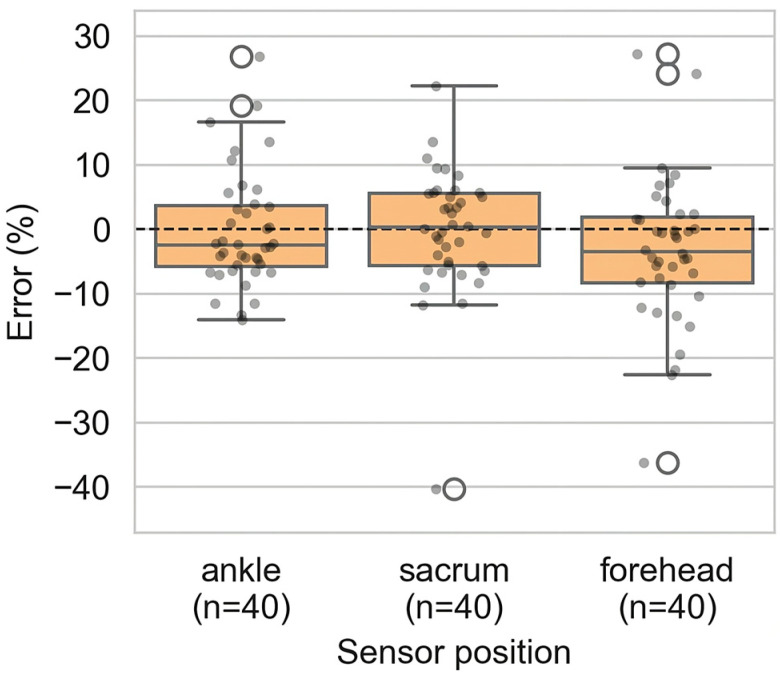
Relative error (%) of fall-height estimates by P2S vs. Kinovea at the ankle, sacrum, and forehead (n = 40). Boxes represent the interquartile range (IQR), the central line the median, whiskers the data range excluding outliers, individual dots correspond to single trials, open circles indicate outliers, and the dashed horizontal line represents zero error.

Fall heights estimated from P2S were consistent with Kinovea across all sensor positions (ankle, sacrum, forehead) as shown in Figure 5.

At the ankle, P2S slightly underestimated fall height (−0.3 ± 8.9%, *p* = 0.061). At the sacrum, the bias was minimal (−0.2 ± 9.7%, *p* = 0.555). At the forehead, a small underestimation was observed (−3.4 ± 11.5%, *p* = 0.095). None reached statistical significance as detailed in Table S1.

### 3.3. Peak Velocity

**Figure 6:**
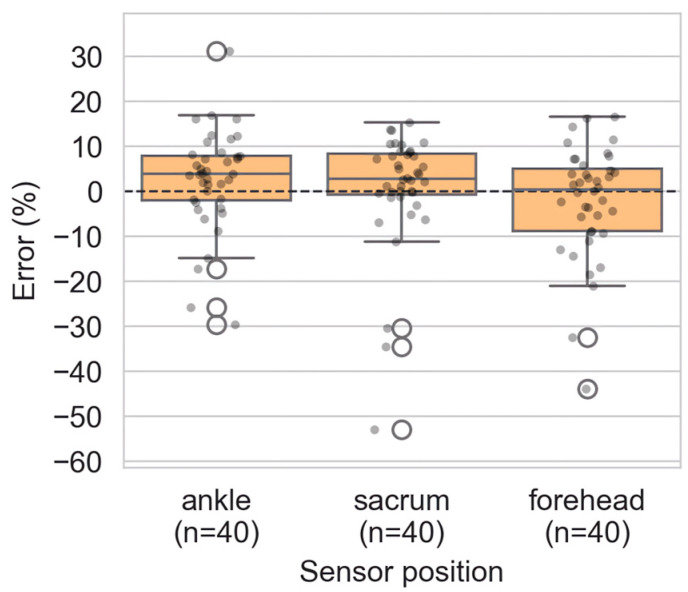
Relative error (%) of peak-velocity estimates by P2S vs. Kinovea at the ankle, sacrum, and forehead (n = 40). Boxes represent the interquartile range (IQR), the central line the median, whiskers the data range excluding outliers, individual dots correspond to single trials, open circles indicate outliers, and the dashed horizontal line represents zero error.

Peak velocities obtained with P2S also showed no significant differences compared to Kinovea for all sensor locations (Figure 6). Maximum velocities reached 7.8 m·s^−1^ (Kinovea) and 6.9 m·s^−1^ (P2S) at the ankle, 7.1 m·s^−1^ (Kinovea) and 6.9 m·s^−1^ (P2S) at the sacrum, and 7.4 m·s^−1^ (Kinovea) and 6.9 m·s^−1^ (P2S) at the forehead.

Figure 6 and Table S2 show that, at the ankle, P2S slightly overestimated velocity (+2.5 ± 11.2%, *p* = 0.051). At the sacrum, the mean bias was small and non-significant (+0.6 ± 13.2%, *p* = 0.063). At the forehead, P2S tended to underestimate velocity (−2.3 ± 12.5%, *p* = 0.590).

### 3.4. Agreement Between P2S and Kinovea

**Figure 7:**
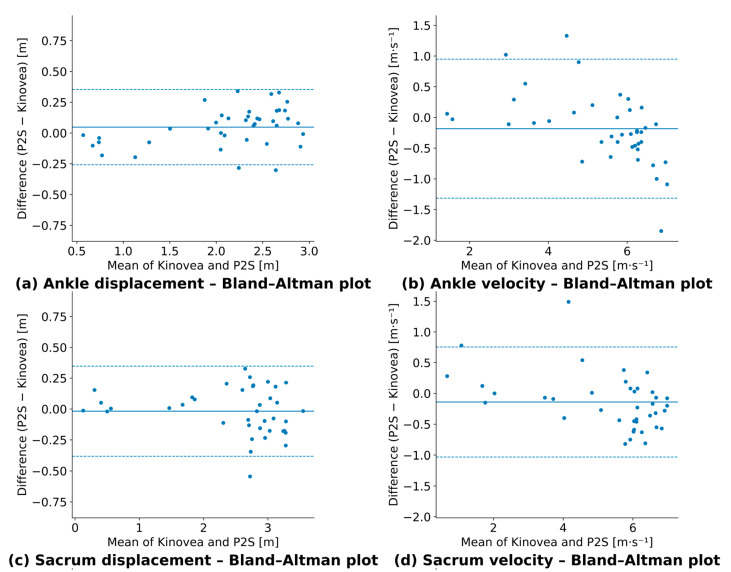

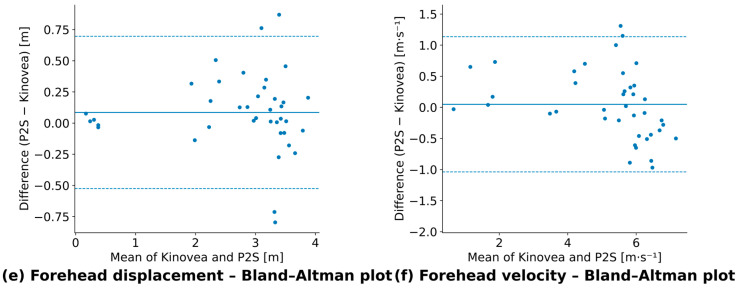
Bland–Altman plots comparing P2S and Kinovea for displacement and velocity across three body segments. Solid line: mean difference; dashed lines: 95% limits of agreement. (**a**) ankle displacement; (**b**) ankle velocity; (**c**) sacrum displacement; (**d**) sacrum velocity; (**e**) forehead displacement; (**f**) forehead velocity.

Intraclass correlation coefficients of Figure 7 confirmed an excellent agreement between P2S and Kinovea for both displacement and velocity across all segments (ICC (2,1) = 0.984–0.989) [38].

Bland–Altman plots (Figure 7) showed small mean biases for displacement (+0.05 to +0.09 m and one slightly negative value of −0.02 m) and velocity (−0.18 to +0.05 m·s^−1^), with 95% limits of agreement ranging approximately from −0.53 to +0.70 m for displacement and −1.31 to +1.13 m·s^−1^ for velocity. These results confirm the strong concordance between P2S and Kinovea (ICCs = 0.96–0.99). Full agreement metrics are provided in Table S3.

### 3.5. Peak Acceleration

#### 3.5.1. Peak Magnitude

**Figure 8:**
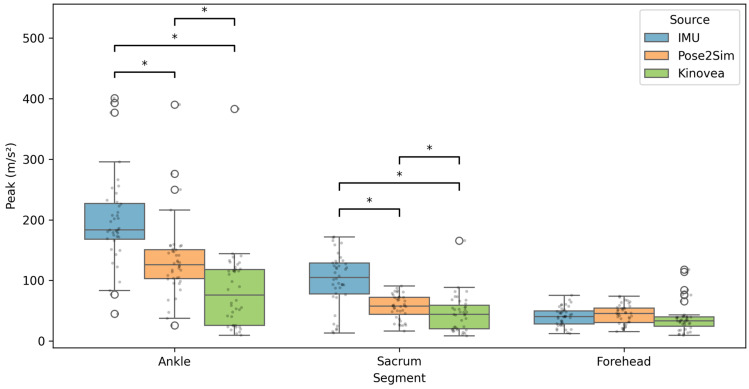
Peak acceleration measured by P2S, Kinovea, and IMUs at the ankle, sacrum, and forehead (n = 39–40). Boxplots display the median (center line), interquartile range (box), and whiskers; filled dots represent individual trials and open circles indicate outliers. Brackets indicate pairwise comparisons. Asterisks denote statistical significance (* *p* < 0.05). For sacrum and forehead, n = 39 because one trial showed no identifiable acceleration peak at those locations (landing impulse mainly concentrated at the ankle).

In contrast with fall height and velocity, peak acceleration showed systematic underestimation by video-based methods compared with IMUs (Figure 8).

As shown in Figure 8, peak-acceleration distributions were wider at the ankle and sacrum than at the forehead, indicating greater trial-to-trial variability and higher impacts at lower-body sensors. This variability is expected because trials were purposely selected across multiple start heights and landing outcomes, leading to a broad range of impact intensities.

At the ankle and sacrum, IMU peak accelerations were significantly higher than both P2S and Kinovea (all *p* < 0.05). At the forehead, no significant differences were observed between methods (all *p* > 0.05). Effect sizes (Cohen’s dz) are reported alongside *p*-values in Table S4.

#### 3.5.2. Peak Width

To further verify the accuracy of P2S in estimating peak accelerations compared to IMUs, we examined the time duration around the fall peak values.

The resulting widths are summarized in Table 2. At the ankle, IMU peaks were markedly wider than P2S (38.0 ± 16.2 ms vs. 21.3 ± 6.7 ms, *p* < 0.001), and at the sacrum they were slightly but significantly wider (24.8 ± 6.2 ms vs. 21.8 ± 6.0 ms, *p* = 0.0373). At the forehead, peak widths were similar between IMU and P2S (17.3 ± 12.8 ms vs. 19.1 ± 5.1 ms, *p* = 0.5528), indicating no systematic difference for this segment.

### 3.6. Impact of IMU Resampling

**Figure 9:**
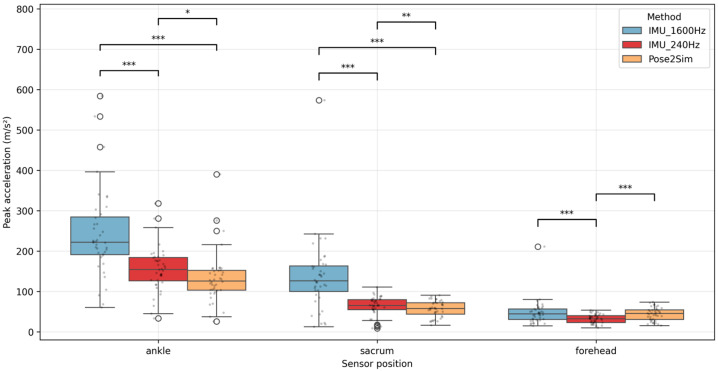
Peak acceleration comparison between IMU (1600 Hz and filtered) vs. IMU (240 Hz and filtered) vs. P2S (240 FPS and filtered) at the ankle, sacrum, and forehead. Boxplots display the median (center line), interquartile range (box), and whiskers; filled dots represent individual trials and open circles indicate outliers. Brackets indicate pairwise comparisons. Asterisks denote statistical significance (* *p* < 0.05, ** *p* < 0.01, *** *p* < 0.001).

A direct comparison between the original IMU signal (1600 Hz) and the same data resampled to 240 Hz confirmed that limited temporal resolution substantially attenuates peak accelerations (Figure 9).

#### 3.6.1. IMU 1600 Hz vs. IMU 240 Hz Comparison

Resampling IMUs from 1600 Hz to 240 Hz significantly reduced peak acceleration measurements across all sensor locations. This attenuation reflects the loss of high-frequency components in short-duration impact transients; consequently, the sharpest peaks (and, when present, multiple closely spaced sub-peaks) may be underestimated or merged after resampling. At the ankle, IMU 1600 Hz recorded peak acceleration of 198.1 ± 75.7 m·s^−2^, while IMU 240 Hz measured 155.3 ± 56.4 m·s^−2^ (−21.6% reduction). Similar patterns were observed at the sacrum (1600 Hz: 100.4 ± 44.6 m·s^−2^, 240 Hz: 63.3 ± 24 m·s^−2^, −37% reduction) and forehead (1600 Hz: 40.3 ± 15.6 m·s^−2^, 240 Hz: 32.2 ± 11.2 m·s^−2^, −20.1% reduction). Importantly, this resampling analysis was used to quantify the technical effect of limited temporal resolution on peak estimation and to enable a fair comparison with video-based methods.

#### 3.6.2. IMU 240 Hz vs. P2S Comparison

When comparing IMU data at 240 Hz with P2S measurements, the values showed closer agreement than with IMU 1600 Hz data (Table S5). At the ankle, IMU 240 Hz measured 155.3 ± 56.4 m·s^−2^ compared to P2S values of 133.2 ± 64.0 m·s^−2^ (−14% difference, *p* = 0.032). At the sacrum, IMU 240 Hz recorded 63.3 ± 24.0 m·s^−2^ versus P2S values of 56.6 ± 20.0 m·s^−2^ (−11% difference, *p* = 0.006). At the forehead, P2S measured 43.8 ± 16.1 m·s^−2^ compared to IMU 240 Hz values of 32.2 ± 11.2 m·s^−2^ (+36% difference, *p* < 0.001).

## 4. Discussion

This study aimed to validate a markerless multi-camera system (P2S) for capturing bouldering fall kinematics in a real gym setting, addressing the need for practical field methods in climbing research. By bridging a gap highlighted by high injury rates in bouldering [1,2,3,4,5], our work builds on recent markerless motion capture advances to assess whether P2S can reliably quantify fall dynamics. The results indicate that P2S accurately reconstructed key measures of fall kinematics, with certain limitations. Segment lengths derived from P2S matched anthropometric measurements, demonstrating good morphological fidelity of the skeletal model. Likewise, fall heights and peak velocities from P2S closely agreed with values from a 2D video reference (Kinovea), showing no significant bias across measurement locations. These findings confirm the validity of P2S for estimating displacement and velocity in climbing falls, extending prior evidence of its accuracy in controlled movements [15,16,17,18,19,20]. Notably, however, P2S underestimated peak impact accelerations compared to high-frequency IMUs, with mean errors on the order of ~40% at the ankle and sacrum. At the head, where actual accelerations were smaller, no significant difference was observed between video-based and IMU measurements. We also observed that complex falls involving a backward roll after the initial foot landing yielded larger errors, likely because rapid body rotations led to self-occlusions that challenged the pose estimation process [39]. Together, these results suggest that P2S can capture global fall kinematics well, but it under-represents the intensity of impacts.

Our findings align with prior validations of P2S in cyclic movements such as gait, running, and cycling, where accurate displacement and velocity estimates have been reported under controlled laboratory conditions [15,16,17,18,19,20]. Extending this validation to the context of bouldering falls demonstrates the robustness of P2S in a more challenging real-condition setting. The strong agreement between P2S and Kinovea for fall height and velocity is consistent with Kinovea’s documented reliability for planar motion analysis in jumping and running tasks [7,8,9] and has even been applied to fall reconstruction scenarios such as experimental e-scooter crashes, where it successfully estimated head displacement and velocity [10]. This suggests that when movements occur largely in a single plane, both 2D and 3D video-based methods provide robust measures of displacement and speed. Beyond our specific setup, systematic reviews and meta-analyses indicate that modern markerless systems can reach accuracy levels comparable to reference systems in gait analysis and other biomechanical applications [39] and are increasingly considered for clinical use [40]. However, Kinovea is restricted to planar motion and, by default, applies a 2nd-order Butterworth low-pass filter with an automatically determined cutoff [34], which can attenuate peak events [31]. Indeed, in our dataset both P2S and Kinovea produced very similar results for fall height and peak velocity with lower acceleration values compared to IMUs, implying that the two video-based methods share common limitations in capturing abrupt dynamics. Prior work using markerless or video analysis in other high-impact scenarios has noted comparable trends: overall kinematics can be measured reliably, but peak forces or accelerations tend to be underestimated or missed [41]. These parallels reinforce that our observations are not unique to climbing falls but reflect general limits of video-based motion analysis for fast events.

The systematic underestimation of impact acceleration by P2S can be explained by fundamental technical factors rather than flaws in the P2S algorithm itself. More broadly, this limitation reflects fundamental constraints of video-based kinematics (finite frame rate and noise amplification by numerical differentiation), not a P2S-specific issue. First, standard video frame rates (240 fps in our case) impose a temporal resolution far lower than that of IMUs (1600 Hz), making it inherently difficult to capture the short-lived spikes of acceleration at impact. Our resampling analysis confirmed that temporal resolution plays a major role: downsampling IMU data from 1600 Hz to 240 Hz reduced peak accelerations by ~20 to 37% [27,28,29]. However, even after accounting for this sampling effect, systematic differences remained. Second, some degree of smoothing must be applied to video-derived trajectories to reduce noise, and this filtering intrinsically blunts the true peaks in the signal [31,32,33]. Additionally, calculating acceleration from positional data requires numerical differentiation, which amplifies any small tracking errors and further diminishes the amplitude of detected peaks [31]. In addition, inconsistencies in joint identification and pose reconstruction remain a known limitation of markerless pipelines, which can further affect the reliability of acceleration estimates [42]. Consequently, IMUs remain the gold standard for high-frequency impact analysis [11,12,13,14], and our findings confirm that even an advanced markerless pipeline cannot fully substitute for IMUs in quantifying impact intensity under the current experimental conditions.

Technical challenges inherent to bouldering falls further exacerbated these discrepancies. As illustrated in Table 1, falls with extensive body rotations (the “roll” falls) presented unique difficulties: the deformable mat surface often obscured ankle joints as feet sank into the material, while impact-induced body rotation caused limbs to cross in front of the torso. These self-occlusions and the compressed landing postures resulted in temporary loss of joint tracking or ambiguous anatomical landmarks, which propagated errors through the kinematic chain. This highlights that highly dynamic movements present a continuing challenge for markerless systems. Recent work has also shown that the magnitude of markerless inconsistencies can be at least as large as soft-tissue artifacts affecting marker-based systems [42]. Nonetheless, ongoing improvements in model fitting, multi-view geometry, and pose estimation algorithms are progressively addressing such cases [15,16,19,20].

Despite these limitations, the practical implications of this work are significant. Our validation demonstrates that markerless video analysis is a viable tool for characterizing global fall kinematics in climbing. P2S provided a detailed picture of each fall’s trajectory, body configuration, and landing posture, enabling objective classification of fall types and identification of potentially risky techniques. Such information is directly relevant for injury prevention efforts in bouldering, where understanding how climbers fall is key to developing safer landing strategies [3,4,6]. Importantly, the markerless approach achieves this without the need for reflective markers or lab-based setups, making it well-suited for real-world gym environments. The full-body kinematics obtained can also be exported to musculoskeletal modeling frameworks for deeper analysis of joint loads and forces [25,26]. On the other hand, our results underscore that high-sampling-rate IMUs are still indispensable for capturing the true magnitude of impact shocks. We recommend a complementary approach in practice: using markerless video to capture the overall movement patterns and fall typology, while employing IMUs at critical locations (e.g., ankles, hips) to measure peak impact accelerations [11,12,13,14]. This fusion of video and sensor data could provide the most comprehensive assessment of bouldering fall biomechanics.

Based on the present validation, P2S alone is appropriate when the research or applied question concerns global fall kinematics, such as fall height, peak velocity, trajectory, body orientation, and fall typology in real-condition gym settings. In contrast, when the objective is to quantify impact intensity (e.g., peak acceleration at the ankle or sacrum, short-duration impact transients), high-sampling-rate IMUs remain indispensable, as video-derived accelerations are intrinsically limited by frame rate, smoothing, and numerical differentiation. In practice, we recommend a hybrid workflow: use markerless multi-camera reconstruction to characterize the whole-body fall kinematics and landing strategy, and deploy IMUs on critical sites (e.g., ankle and pelvis/sacrum) to capture the magnitude of impact peaks. When IMUs are not feasible, higher-frame-rate cameras and optimized filtering may reduce attenuation, but peak impact metrics should be interpreted conservatively.

Finally, some limitations of our study should be acknowledged. The 240-fps camera frame rate and required filtering undoubtedly led to attenuation of peak acceleration values. Improving the temporal resolution (for instance, with >500 fps cameras) would likely enhance the accuracy of video-based acceleration estimates in future studies. Our multi-camera setup consisting of five cameras positioned around the wall did not provide full 360° coverage of the athlete, leading to inevitable blind zones, especially during fast or rotated movements. Additionally, our participant group consisted of adolescent climbers; therefore, generalization to adult or elite climbers should be made with caution. The mean height and body mass of our participants (163.9 cm, 52.8 kg) were lower than normative values reported for a 50th percentile adult male (174.9 cm, 78.6 kg) [43], and such anthropometric differences are known to influence impact kinematics (e.g., peak magnitude and timing). Moreover, age and experience can influence landing strategies (e.g., anticipation of contact, limb stiffness modulation, and intentional rolling or energy dissipation). Together, these factors could affect both the distribution of fall typologies and absolute peak accelerations. Sex-stratified analyses were not included because the sample was small and unbalanced (7 females and 3 males, 25 female falls vs. 15 male falls). Moreover, sex is intertwined with anthropometrics (body mass/height), exposure (fall height; controlled vs. natural trials), and landing strategies (posture/anticipation), making it difficult to isolate a sex effect without overinterpretation; a dedicated study with larger, balanced groups will be required. Even though multiple falls were recorded per participant; analyses were conducted at the trial level. Mixed-effects or repeated-measures models were not applied due to the limited number of participants and the unbalanced number of falls per participant; as a result, treating falls as independent observations may not fully account for within-participant correlation. Furthermore, our dataset mixed natural falls and controlled jumps; although these conditions may differ in anticipatory neuromuscular control, we did not stratify analyses by “natural” vs. “controlled”. Instead, trials were grouped by landing posture (Standing, Loss of balance, Roll), which more directly governs impact mechanics and markerless tracking difficulty (e.g., rapid rotations and self-occlusions). Future studies should explicitly compare anticipated vs. unanticipated falls. Despite these caveats, this study provides a validation of markerless motion capture for climbing falls. In summary, P2S can reliably measure how climbers fall (heights, velocities, body orientations), but not the full intensity of impact; for that, IMUs remain essential. By combining the strengths of both markerless video and wearable sensors, future work can build a more complete understanding of fall mechanics to inform evidence-based injury prevention strategies in climbing.

## 5. Conclusions

P2S, a markerless multi-camera pipeline, was tested for use in bouldering falls performed in an indoor climbing gym. The method reproduced segment lengths with acceptable fidelity and provided fall height and velocity estimates comparable to a 2D video reference. However, it underestimated peak acceleration compared to IMUs, particularly at the ankle and sacrum, while no significant differences were found at the forehead.

These results indicate that markerless video analysis can reliably capture global fall kinematics (height, velocity), whereas IMUs remain essential for quantifying impact intensity. Accordingly, P2S can be used as a standalone tool for fall kinematics and typology, whereas IMUs should be included whenever impact peak quantification is required. This limitation primarily reflects video acquisition constraints (frame rate, restricted camera viewpoints, occlusions), rather than the P2S pipeline itself. Combining both approaches may therefore provide the most comprehensive evaluation of bouldering fall biomechanics and inform future injury prevention strategies.

Future work should explore the use of higher-frame-rate cameras (>500 fps) to improve temporal resolution and capture peak dynamics more accurately. Markerless approaches were also applied to estimate hip impacts in simulated falls among older adults, suggesting potential applications beyond sports [41]. Continuous advances in 3D human pose estimation algorithms are expected to further improve reconstruction accuracy [44], which is consistent with a systematic review showing rapid progress in markerless methodologies [39]. Such approaches may also gain relevance in clinical and rehabilitation contexts, where markerless motion capture already shows promising applications [40]. In addition, alternative strategies such as estimating ground reaction forces directly from 2D pose data could complement the combination of both IMUs and video in future studies [45]. Ultimately, these methodological advances could support the development of safer landing techniques, evidence-based injury prevention strategies in climbing gyms, and safer equipment [3,4,6].

## Figures and Tables

**Table 1:** Morphological fidelity (mean ± SD, in %) of P2S segment reconstructions relative to anthropometric measurements across Standing, Loss of Balance, and Roll conditions, with and without exclusion of extreme errors (>1000%).

Segment (Side)	Standing (15)		Loss of Balance (12)		Roll (13)		Total (40)	
Outliers	With	Without	With	Without	With	Without	With	Without
Upper arm (R)	8.4 ± 61.4	−5.2 ± 12.9	2.9 ± 17.4	−0.7 ± 7.4	42.1 ± 86.3	18.5 ± 35.1	17.6 ± 62.8	4.0 ± 23.6
Upper arm (L)	21.1 ± 54.3	4.0 ± 19.2	4.8 ± 23.3	3.0 ± 17.2	536.9 ± 1804.7	20.0 ± 50.6	61.7 ± 266.5	8.9 ± 23.6
Forearm (R)	12.6 ± 52.8	−1.1 ± 13.3	−11.0 ± 6.2	−11.0 ± 6.2	27.1 ± 71.0	23.3 ± 61.6	9.7 ± 52.3	3.6 ± 38.0
Forearm (L)	20.1 ± 50.7	4.7 ± 20.4	10.6 ± 45.7	−1.3 ± 14.6	535.8 ± 1683.3	7.5 ± 29.8	71.5 ± 270.5	3.6 ± 22.1
Trunk (R)	1.8 ± 17.1	−1.0 ± 14.4	1.0 ± 12.3	1.0 ± 12.3	18.5 ± 58.4	7.0 ± 23.6	7.3 ± 35.6	2.3 ± 17.3
Trunk (L)	7.3 ± 28.8	−0.3 ± 10.5	−3.1 ± 6.5	−3.1 ± 6.5	14.3 ± 48.5	9.0 ± 44.9	6.2 ± 32.6	1.8 ± 26.4
Thigh (R)	7.9 ± 26.3	2.1 ± 11.9	−1.9 ± 8.4	−1.9 ± 8.4	34.9 ± 85.9	13.6 ± 37.7	13.5 ± 53.4	4.5 ± 23.4
Thigh (L)	9.8 ± 13.5	3.9 ± 13.3	4.3 ± 15.3	1.8 ± 8.4	48.2 ± 132.7	13.1 ± 32.2	20.5 ± 78.9	6.2 ± 20.6
Shank (R)	−0.5 ± 21.1	−2.5 ± 13.9	5.5 ± 30.5	−0.3 ± 10.6	35.9 ± 145.6	1.5 ± 25.7	13.0 ± 84.9	−0.5 ± 17.5
Shank (L)	5.4 ± 17.6	2.9 ± 14.7	5.4 ± 11.3	3.8 ± 10.5	47.0 ± 165.6	4.3 ± 19.6	18.9 ± 94.7	3.7 ± 15.0

**Table 2:** Paired *t*-tests comparing IMU vs. P2S peak acceleration width (ms) by segment: mean ± SD and post hoc paired *t*-test *p*-values.

**Segment**	**IMU (Mean ± SD)**	**P2S (Mean ± SD)**	***p* (IMU vs. P2S)**
Ankle	38.0 ± 16.2	21.3 ± 6.7	<0.001
Sacrum	24.8 ± 6.2	21.8 ± 6.0	0.0373
Forehead	17.3 ± 12.8	19.1 ± 5.1	0.5528

## Data Availability

The data presented in this study are available on request from the corresponding author.

## Supplementary Materials

The following supporting information can be downloaded at https://www.mdpi.com/article/10.3390/s26020662/s1, Table S1. Relative error (%) in fall-height P2S versus Kinovea: means, SD, and paired *t*-test *p*-values at the ankle, sacrum, and forehead (negative values indicate underestimation by P2S); Table S2. Relative error (%) in peak-velocity P2S versus Kinovea: means, SD, and paired t-test *p*-values at the ankle, sacrum, and forehead (negative values indicate underestimation by P2S); Table S3. Agreement analysis between P2S and Kinovea for displacement and velocity; Table S4. Peak acceleration (m·s^−2^) by segment and method (IMUs, P2S, Kinovea): mean ± SD and post hoc paired *t*-test *p*-values (Cohen’s dz); Table S5. Paired *t*-tests comparing IMU resampled vs. P2S peak acceleration (m·s^−2^) by segment: mean ± SD and post hoc paired *t*-test *p*-values.

## Abbreviations

The following abbreviations are used in this manuscript:

IMU	Inertial Measurement Unit
P2S	Pose2Sim
RM-ANOVA	Repeated-Measures Analysis of Variance
SD	Standard Deviation
CSV	Comma-Separated Values
fps	Frames per Second
Hz	Hertz
m	meter
cm	centimeter
ms	millisecond
m·s^−1^	Meters per Second
m·s^−2^	Meters per Second Squared
2D	Two-dimensional
3D	Three-dimensional
ICC	Intraclass Correlation Coefficient
LoA	Limits of Agreement
