# Supplementary material for: Validation of a Markerless Multi-Camera Pipeline for Bouldering Fall Kinematics
Source: Sensors (Basel). 2026 Jan 19;26(2):662. doi: 10.3390/s26020662 (PMC12846235; doi:10.3390/s26020662)
Supplement: Supplementary file 1 [file sensors-26-00662-s001.zip › sensors-4064311-supplementary.pdf]

Supplementary Materials:

Table S1. Relative error (%) in fall-height P2S versus Kinovea: means, SD, and paired *t*-test *p*-values at the ankle, sacrum, and forehead (negative values indicate underestimation by P2S)

Error (%)	ankle	sacrum	forehead
**means**	-0.3	-0.2	-3.4
**SD**	8.9	9.7	11.5
***t*-test (p-values)**	0.06149	0.55545	0.09493

Table S2. Relative error (%) in peak-velocity P2S versus Kinovea: means, SD, and paired *t*-test *p*-values at the ankle, sacrum, and forehead (negative values indicate underestimation by P2S)

Error (%)	ankle	sacrum	forehead
**means**	2.5	0.6	-2.3
**SD**	11.2	13.2	12.5
***t*-test (p-values)**	0.05118	0.06293	0.5899

Table S3. Agreement analysis between P2S and Kinovea for displacement and velocity

Segment	Variable	CC (2,1)	Bias	95% LoA (lower–upper)
**Ankle**	Displacement (m)	0.972	+0.05	[−0.26 ; 0.35]
**Sacrum**	Displacement (m)	0.986	-0.02	[−0.38 ; 0.35]
**Forehead**	Displacement (m)	0.958	+0.09	[−0.53 ; 0.70]
**Ankle**	Velocity ($m·s^{-1}$)	0.987	−0.18	[−1.31 ; 0.95]
**Sacrum**	Velocity ($m·s^{-1}$)	0.989	−0.14	[−1.03 ; 0.74]
**Forehead**	Velocity ($m·s^{-1}$)	0.985	+0.05	[−1.04 ; 1.13]

Table S4. Peak acceleration ($m·s^{-2}$) by segment and method (IMUs, P2S, Kinovea): mean ± SD and post hoc paired *t*-test *p*-values (Cohen's dz)

Segment	IMU (Mean± SD)	P2S (Mean ± SD)	Kinovea (Mean ± SD)	*p*(dz) IMU vs P2S	*p*(dz) IMU vs Kinovea	*p*(dz) P2S vs Kinovea
**Ankle**	198.1 ± 75.7	133.2 ± 64.0	83.5 ± 66.4	<0.001 (0.87)	<0.001 (1.42)	0.0007 (0.58)
**Sacrum**	100.4 ± 44.6	56.6 ± 20.0	45.6 ± 30.0	<0.001 (1.43)	<0.001 (1.23)	0.0223 (0.38)
**Forehead**	40.3 ± 15.6	43.8 ± 16.1	38.0 ± 25.3	0.0531 (-0.32)	0.5124 (0.11)	0.1303 (0.25)

Table S5. Paired *t*-tests comparing IMU resampled vs P2S peak acceleration ($m·s^{-2}$) by segment: mean ± SD and post hoc paired *t*-test *p*-values

Segment	IMU 240 Hz (Mean± SD)	P2S (Mean ± SD)	*p* (IMU 240Hz vs P2S)
**Ankle**	155.3±56.4	133.2 ± 64.0	0.03212
**Sacrum**	63.3±24	56.6 ± 20.0	0.00611
**Forehead**	32.2±11.2	43.8 ± 16.1	<0.001
